# *RAS* mutations vary between lesions in synchronous primary colorectal cancer: testing only one lesion is not sufficient to guide anti-EGFR treatment decisions

**DOI:** 10.18632/oncoscience.118

**Published:** 2015-02-09

**Authors:** Mariana Petaccia de Macedo, Fernanda Machado de Melo, Júlia da Silva Ribeiro, Celso Abdon Lopes de Mello, Maria Dirlei Ferreira de Souza Begnami, Fernando Augusto Soares, Dirce Maria Carraro, Isabela Werneck da Cunha

**Affiliations:** ^1^ Department of Molecular Diagnosis, Anatomic Pathology Department, AC Camargo Cancer Center, São Paulo, Brazil; ^2^ Laboratory of Investigative Pathology, CIPE / AC Camargo Cancer Center, São Paulo, Brazil; ^3^ Department of Clinical Oncology, AC Camargo Cancer Center, São Paulo, Brazil; ^4^ Laboratory of Genomics and Molecular Biology, CIPE / AC Camargo Cancer Center, São Paulo, Brazil

**Keywords:** synchronic primary colorectal cancer, colorectal cancer, KRAS, NRAS, anti-EGFR treatment

## Abstract

**Introduction:**

Mutations in *KRAS* and *NRAS* genes are negative predictors of anti-EGFR therapies response in metastatic colorectal cancer. There are few reports on RAS testing in synchronous primary colorectal cancer (SP-CRC) and a lack of recommendations on which tissue should be tested for the mutation in this disease. This study analyzed the *RAS* status of both lesions in SP-CRC patients and in their metastasis.

**Materials and methods:**

DNA was obtained from formalin-fixed-paraffin-embedded tissue, and mutations were analyzed by pyrosequencing.

**Results:**

RAS status was heterogeneous in 6 (75%) of 8 SP-CRC patients between primary lesions. Five showed heterogeneity regarding *RAS* mutational status, and from these, four presented with metastasis: 3 cases (75%) had WT metastatic tissue, and 1 case (25%) had mutated metastatic tissue. One patient showed divergence regarding *RAS* mutation type.

**Discussion:**

*RAS* mutations vary significantly between SP-CRC lesions, and the status of the metastasis is unpredictable. Testing for *RAS* mutations in only 1 of the primary lesions can misguide clinical decisions and hind the predictive potential of anti-EGFR treatment. A more appropriate approach in metastatic SP-CRC is to test the metastatic tissue or both primary lesions for providing more accurate mutation scenery and support more assertive clinical decisions.

## INTRODUCTION

The definition of synchronous primary colorectal carcinoma (SP-CRC) is the existence of more than one primary colorectal carcinoma (CRC) in a single patient1. This condition differs from metastatic synchronous CRC, in which metastasis is diagnosed at the time of the primary tumor [2]. SP-CRC is estimated to account for 3.5% of all CRCs [1]. SP-CRC is more common in men and is associated to predisposing conditions, such as inflammatory bowel disease, hereditary nonpolyposis CRC (Lynch Syndrome), and familial adenomatous polyposis [1].

The prognosis of SP-CRC is unknown [3, 4, 5]. Compared with solitary CRC, SP-CRC is more often associated with right-sided tumors, mucinous histology, and precursor sessile serrated adenoma (SSA). Molecularly, SP-CRC is linked to high microsatellite instability (MSI-H) and concurrent *BRAF* gene mutations [4], although these relationships are controversial [3].

Mutations in *KRAS* are well-established negative predictors of the response to anti-EFGR therapies in the treatment of metastatic CRC [6]. *KRAS* mutations are observed in 35% to 40% of CRCs and arise more often in codons 12 (80%) and 13 (15%) of exon 2 [7, 8, 9] and to a lesser extent in codons 61, 117, and 146 [7, 8, 9]. Unsual *KRAS* mutations affecting more than 1 codon and insertions have also been reported [10, 11]. Recent studies have shown that CRC patients with tumors that harbor *NRAS* gene mutations also have poorer response rates to EGFR inhibitors compared with those with wild-type *NRAS* [12]. *NRAS* mutations, present in approximately 5% of CRC tumors, are less frequent than *KRAS* mutations [13] and also developed most often in codons 61, 12, and 13. Concomitant mutations in *KRAS* and *NRAS* are a rare finding [12].

Thus, testing for *KRAS* and *NRAS* mutations is necessary before anti-EGFR therapies are initiated in CRC patients [12, 14, 15]. Concerns have been raised since the *KRAS* mutation testing recommendations were issued regarding the ideal tissue that should be examined. The concordance of *KRAS* status between primary and metastatic CRC tissue in the same patient varies significantly, with heterogeneit*y* ranging from 0% to 31% but tending to be low [16]. Studies that compared CRC biopsies before and after neoadjuvant therapy did not report any differences regarding KRAS status [17, 18, 19], nor did studies that compared biopsy and resection specimens in CRC [20, 21, 22].

As a result, an issue has arisen regarding patients with more than one primary lesion: should *RAS* mutations be tested in both lesions? In the daily routine of a molecular pathology laboratory, facing that situation is not unusual, especially for those that perform high-volume *RAS* mutation testing.

The aim of this study was to analyze *KRAS* and *NRAS* mutational status in both lesions of SP-CRC patients as well as the metastatic tissue and determine the necessity of testing for both lesions in order to provide more precise information for supporting clinical decision.

## RESULTS

### Clinical and pathological data

We retrieved 8 cases with SP-CRC from our molecular pathology laboratory records. The 8-patient series comprised 5 males (62.5%) and 3 females (37.5%), and the mean age was 71.5 years. All patients presented with 2 synchronous invasive CRC lesions at the time of the surgical resection (7 patients) or biopsy (1 patient). Five patients show lymph node (LN) metastasis, 4 of whom had additional systemic metastasis (patient 1-liver, pleural and abdominal; patient 2-lung and brain; patient 5-lung; and patient 8-liver). See Table 1 for their clinical and pathological data.

**Table 1:** Clinicopathological data, mismatch repair protein and RAS status of Synchronous primary Colorectal Carcinomas

Patient	Age	Gender	TU location	TU size	Stage	IHQ MMR Loss	RAS Status	Metastasis	Other information
1	63	M	R (asc)	5,0 cm	T3	Not tested	KRAS c.34G>A (p.G12S)	Reg Ascending LN & metachr Liver: KRAS c.34G>A (p.G12S)	None
			L (desc)	2,5 cm	T3	Not tested	KRAS c.35G>A (p.G12D)		
2	74	M	NS	5,8 cm	T3	No loss	KRAS c.35G>A (p.G12D)	Paraaortic LN & metachr Brain & metachr Lung: KRAS c.35G>A	None
			NS	2,9 cm	T2	MLH-1/PMS2	Wild type		
3	83	F	R (asc)	2,8 cm	T2	MLH-1/PMS2	Wild-type	Reg LN WT	None
			R (asc)	1,2 cm	T1	MLH-1/PMS2	Wild type		
4	83	M	R (cecum)	10,0 cm	T3	MLH-1/PMS2	KRAS c.35G>T (p.G12V)	Reg LN WT	Multiple CBC, RCC
			R (trans)	3,7 cm	T1	MLH-1/PMS2	Wild type		
5	79	M	L (desc)	3,5 cm	T3	No loss	KRAS c.35G>A (p.G12D)	Synchr lung WT	Prostate Cancer
			L (desc)	3,0 cm	T3	No loss	Wild type		
6	64	F	R (transv)	2,5 cm	T3	MSH2/MSH6	Wild-type	None	HNPCC with Kidney and Bladder Cancer
			R (transv)	1,6 cm	T1	MSH2/MSH6	KRAS c.38G>A (p.G13D)		
7	63	F	R (asc)	Biopsy	NA	NA	KRAS c.35G>T (p.G12V)	liver (clinicaly)	None
			R (transv)	Biopsy	NA	NA	KRAS c.35G>T (p.G12V)		
8	69	M	L (desc)	5,5 cm	T3	NA	NRAS c.182A>T (p.Q61L)	Reg LN & synchr liver WT	None
			L (desc)	4,0 cm	T3	NA	Wild type		

R= right sided, L= left sided, LN= lymph node, &= and; Stage=TNM; Tumor size = greatest diameter; IHQ= immunohistochemistry; MMR= Mismatch Repair Protein;

CBC= basal cell carcinoma of the skin, RCC= renal cell carcinoma, NA= not tested; WT= Wild Type for *KRAS* and *NRAS;* ASC= ascending; DESC= descending; TRANSV= transverser; NS= not specified; TU= tumor; REG= regional; SYNCHR= synchronous metastasis; METACHR= metachronous metastasis

### Molecular pathology: KRAS mutational analysis of synchronous carcinomas

Of the 16 primary tumor samples in the 8 patients, 7 had wild-type *RAS* and 9 had mutated *RAS* samples. *KRAS* mutation was the most frequent (8 of 9 mutations, 88%). *KRAS* codon 12 was the most frequently mutated codon (7 of 9 mutated samples, 77%). Three mutations were noted: 3 cases of c.35G>A in *KRAS* codon 12 (p.G12D), 3 cases of c.35G>T (p.G12V) in *KRAS* codon 12, and 1 case of c.34G>A (p.G12S). A *KRAS* mutation in codon 13 (c.38G>A [p.G13D]) was observed in 1 patient, and a *NRAS* mutation in codon 61 (c.182A>T [p.Q61L]) detected in 1 patient. There were no mutations in *KRAS* codons 61, 117, or 146 or in *NRAS* codons 12, 13, 117, or 146.

*RAS* mutations were conflicting in 6 (75%) of the 8 SP-CRC patients analyzed in this study. Conflicts were regarding RAS status and RAS mutation type. Five patients (83% and 62.5% of the heterogeneous or whole study group, respectively) (patients 2, 4, 5, 6, and 8) had 1 lesion with wild-type (WT) *RAS* and 1 lesion with mutated *RAS*; both lesions in the remaining patient (patient 1) harbored a mutation in *KRAS* codon 12 c.35G>A (p.G12D) and c.34G>A (p.G12S), respectively. Of the 2 cases that showed no heterogeneity with regard to *RAS* mutation between both primary CRC lesions, patient 3 had WT RAS in both lesions, and patient 7 had the c.35G>T *KRAS* mutation in both lesions.

Seven of the 8 patients had LN or systemic metastasis. We noted several profiles of LN and systemic metastatic tissue in patients with heterogeneous *RAS* mutation status in the primary lesions. Of the 4 metastatic cases with both WT and mutated *RAS* status, 3 (75%) (patients 4, 5, and 8) and 1 (25%) (patient 2) resulted in the metastatic tissue *RAS* WT and mutated, respectively. In the patient with disparate *RAS* mutations in the primary lesions (patient 1), both the LN and liver metastases had the same *KRAS* c.34G>A (p.G12S) mutation. See Table 1 for *RAS* mutational data.

## DISCUSSION

In this study, we evaluated the *RAS* mutational status of both lesions in 8 patients with SP-CRC and found that *RAS* mutations are commonly heterogeneous between SP-CRC lesions.

The rate of heterogeneity between lesions was 75% with regard to *RAS* mutational status and type. Although some studies have indicated that specific *KRAS* mutations respond to EGFR inhibitors [24], specially p.G13D, *RAS*-mutated tumors generally fail to respond to anti-EGFR treatment, regardless of the nucleotide substitution. If we consider only cases with heterogeneity in *RAS* mutational status (WT and mutated), 62.5% of SP-CRC cases showed clinically relevant heterogeneity of RAS mutational status between primary tumors.

Previous studies have reported molecular heterogeneity of both lesions in SP-CRC. Eguchi and colleagues [25] analyzed p53 mutations in both lesions of 16 SP-CRC and found that 7 patients harbored a p53 mutation in only 1 lesion. In 9 patients, both lesions were mutated, but the mutations always differed between lesions from the same patient. Thus, regarding the p53 mutational status in SP-CRC, the authors found no concordance in p53 mutation status between lesions, suggesting that the synchronous tumors had a multicentric, not monoclonal, origin. Another group [5] showed that the pattern of CpG island methylation was concordant in synchronous cancer pairs in the same location in the colon (proximal-proximal) and colorectum (distal-distal) but not in tumor pairs in differing locations (eg, 1 proximal cancer and 1 distal cancer).

Previous studies have reported a significant percentage of discordance in *KRAS* mutational status between both lesions in SP-CRC. Balschun et al. studied 20 patients with SP-CRC for mutations in *KRAS*, *NRAS*, *PIK3CA,* and *BRAF*. *KRAS* mutations were discordant between synchronous lesions in 6 patients: 3 patients had mutated versus wild-type *KRAS,* and 3 patients had disparate mutation types in the synchronous lesions. *NRAS* status was heterogeneous in 1 patient with 4 primary lesions, only 2 of which harbored an *NRAS* mutation. They also tested the metastatic tissue and reported the ability to predict the origin of a metastasis by comparing the type of mutation between primary lesions [26].

Ogino et al. analyzed 6 SP-CRC patients regarding MSI, *KRAS* and *BRAF* status, and successful sequenced *KRAS* gene of 5 pairs of lesions. They found 3 out of 5 pairs to be discordant with regard to *KRAS* mutation (60%). Two cases showed discordance regarding *KRAS* status (WT versus mutated) and one case showed different types of mutation between paired lesions. Metastatic tissue was not tested. Further, they noted discordance for *BRAF* mutation status between paired lesions (p.V600E and WT status) and MSI status in 1 patient [27].

Konishi et al. evaluated 27 synchronous CRC cases and found 10 patients with discordance regarding *KRAS* mutational status (wild-type versus mutated). The authors did not report any data regarding the type of mutation, thus, the discordance rate might be underestimated if we consider the possibility that *KRAS* mutation type differed between lesions [28].

Bae et al. studied 98 lesions from 46 patients with SP-CRC and showed that *KRAS* mutation rates did not differ statistically between synchronous and solitary CRC, and also stated that *KRAS* and *BRAF* mutation status were not concordant in either of the synchronous pairs [29].

Koness et al. compared *KRAS* mutations in 15 SP-CRC patients and found 7 cases with differences in *KRAS* mutational status between paired tumors but did not show data regarding the type of mutation. Of the 8 cases with similar KRAS status, 1 had a mutation in both lesions [30].

One group [31] compared *RAS, BRAF, PIK3CA*, and TP53 status between 84 pairs of primary CRC and liver metastases and found a concordant rate of 97.6%, 98.8%, and 92.8% for RAS/BRAF, PIK3CA, and TP53, respectively. Regarding the 2 discordant *KRAS* mutation cases, 1 case was actually SP-CRC operated in different times with liver metastasis, and the second case was a patient with mucinous CRC and nonmucinous liver metastasis, with no additional clinical information, which were demonstrated to have developed from different primary lesions.

Collectively, our data and those of previous studies have shown that *KRAS* and *NRAS* mutations vary widely between SP-CRC lesions and that the status of the corresponding metastasis is unpredictable. Testing for *KRAS* mutation in only 1 of the primary lesions in SP-CRC might yield an incomplete profile on *KRAS* and *NRAS* mutation status and can misguide the clinical decision with regard to anti-EGFR treatment.

The best approach to guide anti-EGFR treatment decision in SP-CRC with metastatic disease would be to test the metastatic tissue for *KRAS* and *NRAS* mutations, because it is not possible to be certain which primary SP-CRC lesion led to the metastasis without any additional study. In clinical scenarios of impossibility in obtaining the metastatic tissue, or if patients present with multiple metastases and examining all metastatic sites is not suitable, or for SP-CRC cases in a routine molecular pathology laboratory with no additional clinical information, both primary lesions should be tested.

## MATERIAL AND METHODS

### Study population and histopathological features

Study participants were drawn from an institutional database between 2009 and 2014 and comprised patients of both genders and of all ages with a diagnosis of SP-CRC who were operated or biopsied on at AC Camargo Cancer Center, São Paulo, Brazil, and those who were being followed at our institution after tumor resection by an outside service and had their slides reviwed by our service. The SP-CRC cases in this study had invasive CRC lesions in the same surgical specimen. Pathological data were retrieved from the surgical pathology reports. The tumors were staged per the TNM, 7^th^ edition [23].

### Tissue samples and DNA isolation

Five 5-μm sections from formalin-fixed paraffin-embedded tissue (FFPET) blocks of 1 tumor area of both invasive adenocarcinomas and the metastatic tissue were obtained from the paraffin block. Posterior deparaffinization was performed, and tumor samples were obtained by scraping the neoplastic tissue from the glass slide (macrodissection). The representative tumor area of each case was selected by experienced pathologist, and the minimum of 30% of tumor cell in each selected area was necessary to consider a case suitable for DNA extraction. Genomic DNA was isolated using the QIAamp Kit (Qiagen).

### *KRAS* and *NRAS* mutation analysis

*KRAS* and *NRAS* mutations were analyzed in 1 area of both lesions in SP-CRC patients, as well as in the LNs and systemic metastatic lesions. First, *KRAS* codons 12 and 13 were tested, and if they were wild-type, mutations in *KRAS* codon 61 and *NRAS* codons 12, 13, and 61 were examined. If the sample remained wild-type for the tested codons, then, *KRAS* and *NRAS* codons 117 and 146 were analyzed.

Mutations were evaluated by pyrosequencing per the manufacturer's instructions [*KRAS* PyroMarkTM Q24 kit, NRAS Pyro Kit, RAS extension KIT (Qiagen)]. Ten microliters of biotinylated PCR product was conjugated to streptavidin-sepharose beads (GE Healthcare) per a standard protocol for single-strand preparation. Pyrosequencing was performed using the PyroMarkTM Gold Q24 reagent kit (Qiagen). A cutoff value of 5% was used to define a case as positive.
